# Supplementary material for: Deletion of wfs1 Impairs Oligodendrocyte Precursor Cells Dorsal Distribution and Myelination Through the wfs1-hmgcs1 Axis in Zebrafish
Source: Biology (Basel). 2026 Aug 16;15(16):1402. doi: 10.3390/biology15161402 (PMC13510151; doi:10.3390/biology15161402)
Supplement: Supplementary file 1 [file biology-15-01402-s001.zip › File S1-Transcriptome sequencing data and differentially expressed genes (DEGs) in wfs1-- zebrafish at 5 dpf/2.KEGG/wfs1KOvsWT/DOWN/src/dre04530.html]

dre04530


- dre:792828

- Down regulated genes

792828(-4.67911285540239)

- dre:793158

- Down regulated genes

793158(-6.94943122774273)

- dre:436718

- Down regulated genes

436718\_1(-6.85571323128554)

- dre:792699

- Down regulated genes

792699(-1.84389282152821)

- dre:559392

- Down regulated genes

559392(-2.91317811245432)

- dre:57934

- Down regulated genes

57934(-1.25539807424297)

- dre:100536545

- Down regulated genes

100536545(-1.61631046560488)

- dre:556298

- Down regulated genes

556298(-1.32024152429363)

- dre:57934

- Down regulated genes

57934(-1.25539807424297)

- dre:286739

- Down regulated genes

286739\_1(-2.60068066374091)

- dre:569084

- Down regulated genes

569084\_1(-2.40277205964249)
- dre:81585

- Down regulated genes

81585(-1.74514319517014)
- dre:60635

- Down regulated genes

60635\_1(-1.07799587140666)
- dre:110438642

- Down regulated genes

110438642(-3.83724028917273)

- dre:436718

- Down regulated genes

436718\_1(-6.85571323128554)

- dre:793158

- Down regulated genes

793158(-6.94943122774273)

- dre:445305

- Down regulated genes

445305\_1(-1.98867996431405)

- dre:57934

- Down regulated genes

57934(-1.25539807424297)

- dre:436718

- Down regulated genes

436718\_1(-6.85571323128554)

- dre:797651

- Down regulated genes

797651(-1.27962292749959)

- dre:797651

- Down regulated genes

797651(-1.27962292749959)

- dre:569084

- Down regulated genes

569084\_1(-2.40277205964249)
- dre:81585

- Down regulated genes

81585(-1.74514319517014)
- dre:60635

- Down regulated genes

60635\_1(-1.07799587140666)
- dre:110438642

- Down regulated genes

110438642(-3.83724028917273)

- dre:569084

- Down regulated genes

569084\_1(-2.40277205964249)
- dre:81585

- Down regulated genes

81585(-1.74514319517014)
- dre:60635

- Down regulated genes

60635\_1(-1.07799587140666)
- dre:110438642

- Down regulated genes

110438642(-3.83724028917273)

- dre:569084

- Down regulated genes

569084\_1(-2.40277205964249)
- dre:81585

- Down regulated genes

81585(-1.74514319517014)
- dre:60635

- Down regulated genes

60635\_1(-1.07799587140666)
- dre:110438642

- Down regulated genes

110438642(-3.83724028917273)

- dre:569084

- Down regulated genes

569084\_1(-2.40277205964249)
- dre:81585

- Down regulated genes

81585(-1.74514319517014)
- dre:60635

- Down regulated genes

60635\_1(-1.07799587140666)
- dre:110438642

- Down regulated genes

110438642(-3.83724028917273)

- dre:792828

- Down regulated genes

792828(-4.67911285540239)

- dre:445305

- Down regulated genes

445305\_1(-1.98867996431405)

- dre:57934

- Down regulated genes

57934(-1.25539807424297)

- dre:792699

- Down regulated genes

792699(-1.84389282152821)

- dre:337226

- Down regulated genes

337226(-2.98693262894109)

- dre:793158

- Down regulated genes

793158(-6.94943122774273)

- dre:559392

- Down regulated genes

559392(-2.91317811245432)

Close
